# Sustaining the momentum for global cancer research and career development in the COVID-19 era: Lessons and challenges

**DOI:** 10.7189/jogh.13.03010

**Published:** 2023-04-14

**Authors:** Jonah Musa, Francis A Magaji, Maryam J Ali, Mark Okolo, Olugbenga A Silas, Godwin E Imade, Stefan J Green, Supriya D Mehta, Lifang Hou, Robert L Murphy

**Affiliations:** 1 Department of Obstetrics and Gynecology, College of Health Sciences, University of Jos, Jos, Plateau State, Nigeria; 2 Department of Preventive Medicine, Division of Cancer Epidemiology and Prevention, Feinberg School of Medicine, Northwestern University, Chicago, Illinois, USA; 3 Center for Global Oncology, Institute for Global Health, Feinberg School of Medicine, Northwestern University, Chicago, Illinois, USA; 4 Department of Medical Microbiology, College of Health Sciences, University of Jos, Jos, Plateau State, Nigeria; 5 Department of Anatomic Pathology and Forensic Medicine, College of Health Sciences, University of Jos, Jos, Plateau State, Nigeria; 6 Genomics and Postgraduate Core Facility, College of Health Sciences, University of Jos, Jos, Plateau State, Nigeria; 7 Genomics and Microbiome Core Facility, Rush University, Chicago, Illinois, USA; 8 Division of Epidemiology and Biostatistics, School of Public Health, University of Illinois at Chicago, Chicago, Illinois, USA; 9 Department of Epidemiology and Biostatistics, RUSH University, Chicago, Illinois, USA; 10 Robert H. Lurie Comprehensive Cancer Center and Department of Preventive Medicine, Feinberg School of Medicine, Northwestern University, Chicago, Illinois, USA; 11 Division of Infectious Diseases, Department of Medicine, Feinberg School of Medicine, Northwestern University, Chicago, Illinois, USA; 12 Robert J. Havey MD, Institute for Global Health, Northwestern University, Chicago, Illinois, USA

Cervical cancer is the fourth commonest cancer affecting almost 600 000 women annually worldwide with over 300 000 deaths [1]. Over 70% of these cases are in sub-Saharan Africa with correspondingly high mortality due to late presentation of cases and poor infrastructure for treatment of invasive cancer. Yet, this is one cancer that the natural history is well-known with precancerous conditions that are detectable and treatable when identified through screening. Cervical precancer and invasive cervical cancer is entirely attributable to a sexually transmissible viral infection, the high-risk human papillomavirus (HR-HPV) [2]. A previous report of the International Agency for Research on Cancer (IARC) on the burden of human papillomavirus disease in Nigeria, estimates that over 53 million women in Nigeria are at risk of invasive cervical cancer (ICC); and 14 089 new cases of ICC occur annually which lead to 8240 deaths [3].

The call for action by the World Health Organization (WHO) to eliminate cervical cancer as a public health problem expects member countries to vaccinate 90% of young girls before age 15; screen at least 70% eligible women with a high performance cervical precancer screening test, and provide effective treatment for 90% of women screened with precancerous lesions [4]. Unfortunately, most countries in sub-Saharan Africa including Nigeria have no national programme for HPV vaccination and the coverage for opportunistic cervical precancer screening is reaching less than 9% of women at risk [3]. Therefore, as health policy makers and national governments give priority attention and investment for primary and secondary prevention of this cancer with available effective vaccines and high performing screening test respectively, researchers in the field of preventive oncology must continue to invest in understanding factors that play important roles in cervical cancer development and translate such knowledge to development of novel behavioural or therapeutic tools for prevention and early detection of precancer.

To foster this goal, the National Institutes of Health and the Fogarty International Center (NIH/FIC) have supported training and development of capacity of early career researchers in low-and middle-income countries (LMICs) with the vision that such trained researchers can advance biomedical research in their countries and contribute to finding solutions to locally relevant health problems [5,6]. One of this training opportunity for career development of researchers in LMICs is the “Emerging Global Leader” award otherwise known as the K43 career development award [7]. The primary author of this paper is a beneficiary of the K43 international research career development award.

In the fall of 2019, the FIC K43 program provided funding award to Dr Jonah Musa from the University of Jos, Nigeria (K43TW011416; PI: Musa, J) to advance his research career as an LMIC emerging global cancer researcher focusing on understanding vaginal microbiomes and community state types in a spectrum of cervical precancer and invasive cervical cancer in Jos, Nigeria with co-mentors in Chicago, USA. The goal of this career research is to develop research career in cervical cancer prevention through conduct of the research project aims and acquisition of specific career enhancing skills towards transition to research independence at the end of the award.

Few months into this award, the world was literally shut down by the SARS-COV-2 (COVID-19) pandemic making it impossible for keeping track with in-person career development activities and interrupted the routine interaction in the health care system including enrolments of potential research participants. This unexpected global challenge requires resilience, focus, support, and self-motivation to keep up the momentum and make the utmost gains expected for the research and career development. This paper provides perspectives of an emerging global cancer research leader on sustaining the momentum for such career enhancing research activities in the COVID-19 pandemic era.

## THE PROCESS OF STARTING THE PROJECT POST AWARD

The primary goal of the career research development award is to develop the research skills of the investigator to advance to research independence. This is expected to be accomplished through research with the support of a mentoring team/collaborators who have more experience in the field to provide support and guidance throughout the period of the project. Immediately after receiving the “notice of award”, my team of mentors/collaborators were immediately informed, and we initiated regular start-up calls to plan on how to implement the research project and keep up with the career enhancing courses/workshops as outlined in the career development plan. Within the first quarter of the award (September to December 2019), Dr Musa was able to travel for an in-person planning meeting in Chicago with his co-mentors and collaborators. During the two-week meeting, Dr Musa made excellent progress in understanding the standard operating procedures for sample collection, processing, preservation for subsequent microbiome DNA extraction for microbiome sequencing required for this project. We also finalized on the career enhancing courses at the University of Illinois in Chicago School of Public Health. At the end of the first visit, Dr Musa returned to Jos Nigeria in December 2019 with the required consumables for sample collection for the project. The success of this visit was facilitated by a strong team of mentors who were very committed to the goals of the project and the success of his career as outlined in the career development plan.

Biomedical research involving human subjects as in this project requires the review and approval of the protocol by the research and health ethics committee of our institution. The research protocol, the informed consent documents, and the standard operating procedure documents were submitted to the health and research ethics committee of the Jos University Teaching Hospital (JUTH), Jos, Nigeria. This was reviewed and approved (JUTH/DCS/ADM/127/XXIX/1546) for the commencement of participants enrolment for this project at Jos.

As soon as the protocol was approved by the institutional ethics committee of JUTH, the principal investigator had to fine tune and adapt relevant enrolment documents and select a team of research collaborators within the home institution to ensure successful implementation of the research project at Jos Nigeria. To achieve this and with the counsel and guidance of the US-based co-mentors, a two-day protocol training workshop was convened for all team members comprising: medical microbiologist, gynecologic pathologist, laboratory and genomics scientist, cervical cancer screening and colposcopy nurse, data scientist and REDCap database manager. During the training workshop, the relevant standard operating procedures were presented, and opportunities were given for all team members to ask clarifying questions and make contributions in making the study flow easier for the participants and team. This workshop helped in bringing every member of the team on the same page and created a sense of ownership of the project and the need to remain committed to achieving the goals of the research. The protocol training/meeting with team members created a strong sense of commitment to the research project and contributed to the team support and resilience in keeping on with enrolment procedures and targets even in the face of the COVID-19 pandemic. We also learned great lessons in designing and using the “pink appointment card” to enhance compliance with follow up visits in the longitudinal aim of the project. The pink card has scheduled dates for 12- and 24-month visits with the phone number of the principal investigator for questions and to improve communication and rescheduling of visits for unforeseen circumstances.

## THE CHALLENGES OF THE COVID-19 PANDEMIC

**Figure 1:**
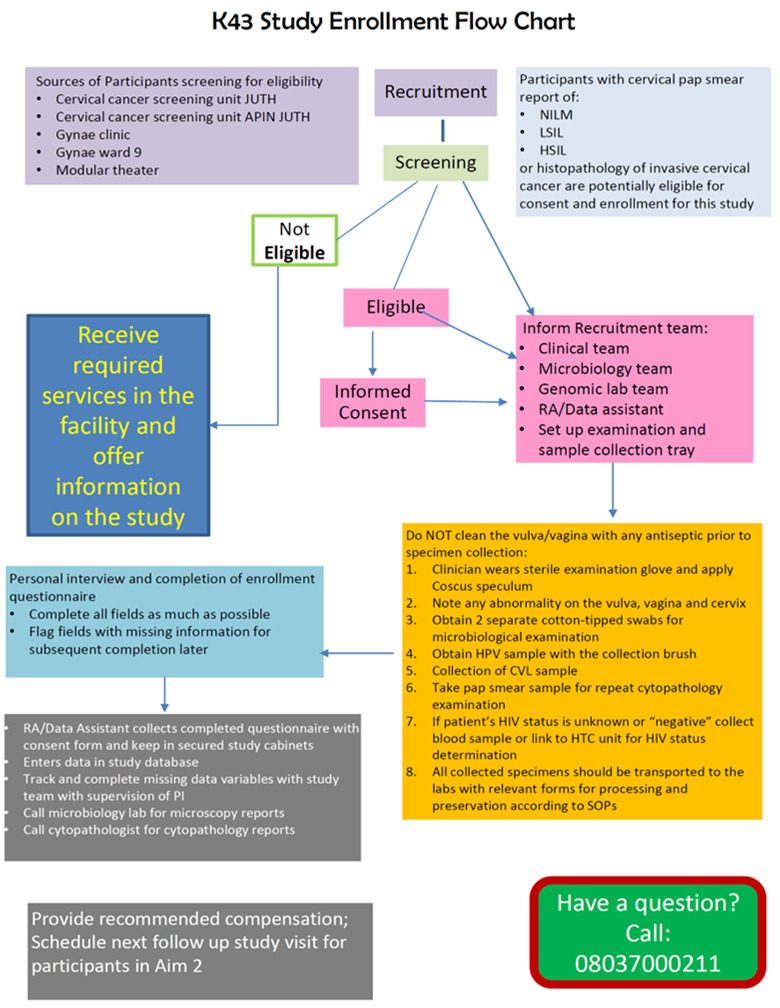
Vaginal microbiome in cervical intraepithelial neoplasia and cancer project flow in Jos, Nigeria.

The COVID-19 pandemic and entire year of 2020 cannot be forgotten soon enough. The lockdowns, the scare and fear of death from the virus brought untold uncertainties, perils, and huge challenges to the health care system in general and affected several research endeavours that depended on interaction with research participants. Patients feared coming to the hospital even for essential services, let alone, cervical cancer screening which in our setting is opportunistic. However, as mentioned earlier, team resilience and support helped in sustaining the momentum for screening and we made tremendous enrolment progress amidst these challenges. Our oncology clinical team were motivated by the understanding that cervical cancer is an emergency, and we kept up with both screening, diagnostic evaluation, and treatment of new cases of cervical cancer while filling up the enrolment numbers into the research project arms. **Figure 1** shows the enrolment flowchart for the project and **Table 1** shows the enrolment summaries for the cross-sectional and longitudinal aims of the project. A screenshot of the enrolment data collected and managed using the Research electronic data capture (REDCap) [8] tools hosted by the University of Jos, Nigeria is illustrated in this viewpoint’s Photo.

**Table 1:** Summary of enrolment for the cross-sectional and longitudinal arms of the project

Cross-sectional aim (cervical pathology)	Enrolment target (n)	Enrolled (n)	Remark
NILM	25	26	On target
LSIL	25	36	Above target
HSIL	25	46	Above target
ICC	25	31	Above target
**Longitudinal aim (low-grade cervical dysplasia)**	**Baseline**	**12-months**	**24-months**
LSIL (50 HIV+)	53	36	4

NILM – negative for intraepithelial lesion or malignancy, LSIL – low-grade squamous intraepithelial lesion, HSIL – high-grade squamous intraepithelial lesion, ICC – invasive cervical cancer, HIV – human immunodeficiency virus

## SUSTAINING MOMENTUM THROUGH VIRTUAL LEARNING

One of the great lessons of the COVID-19 pandemic is the effectiveness of impacting learning, skill acquisition and mentoring support through the virtual platform. Zoom has changed the way we approach interaction and has virtually wiped away barriers in interpersonal communications, instructions, and learning irrespective of distance. During the pandemic, Dr Musa was able to participate in virtual conferences, successfully completed online career enhancing courses and made successful progress with study enrolment. One paradoxical benefit of the COVID-19 travel restrictions is the gain in more time to be physically present onsite for screening and enrolment of research participants. In my experience, the lost opportunities for travels paid off in accruing more research participants without losing much because of the virtual learning opportunities. This experience has been reported in a previous publication [9]. **Table 2** provides a summary of the career enhancing courses, workshops and conferences successfully completed during the COVID-19 travel restrictions and lockdowns.

**Table 2:** Summary of career enhancing activities/courses/workshops attended virtually

Course/workshop/conference	Key areas covered	Institution/organization	Dates	Duration	Career goal(s) achieved
International conference of the Science of Team Science	Understanding diversity and team science in advancing scientific inquiry and discoveries	International Society of the Science of Team Science annual meetings	May 2020 and June 2021	Four days	Scientific and academic leadership; project management, developing competitive grant proposals
Principles of Preclinical Translational Science: a Case study from Cancer Drug Discovery and Development (MEDI 501)	Understanding the difference between translational research and translational science; inter-agency and team-based collaborations necessary for scientific discovery	National Institutes of Health (NIH) Foundation for Advancing Education in Sciences (FAES); NCATS	Summer 2021	Seven weeks virtual course	Team science and translational research process
Genetics in Epidemiology (EPID 520)	Basic genetic concepts, design, analyses, interpretation of genetic data to ethical and legal issues in genetic epidemiology	UIC School of Public Health, Chicago	August 2020 to December 2020	12 weeks	Knowledge in genetic epidemiology including microbiome and omics data analyses, ethics and legal issues in genetic research
Introduction to Bioinformatics: Theory and application: Bioinformatics 082	Hands-on virtual workshop including: introduction to bioinformatics, databases; protein databases; sequence comparison; remote homology searches; evolutionary analysis, and introduction to GALAXY project	NIH/FAES	Four-days intensive virtual workshop, June 2021	Four-days	Knowledge and skills in bioinformatics for genomics data analysis
Leadership Strategies in Biomedical Sciences: Tech 498	Management theories, case presentation, power and leadership, decision making in management, communication, power and negative feedback	NIH/FAES	Virtual workshop with case studies, readings, and group discussions	Seven-weeks course (August to October 2021)	Leadership and management for effective team performance in biomedical research
Introduction to Cancer Biology IMMU 101	Hallmarks of cancer; understanding cell cycle, pathways and targets for cancer drug development	NIH/FAES	Virtual presentations; case discussions; and weekly assignments	Summer 2021	Knowledge on the molecular basis for cancer development and pathways for drug targets
Longitudinal data analysis on STATA	Understanding longitudinal data and modelling time-constant and time-varying covariates	Statistical Horizon	Four-day intensive workshop	August 2022	Skills in multivariate statistical analyses of panel data
Responsible Conduct of Research	Human subject research; informed consent, legacies in scientific racism, research in resource poor settings, ethics of big data, collaboration and authorship; racism	University of California San Francisco (UCSF)	Seven weeks virtual course	July 18-September 2, 2022	Knowledge and skills in responsible conduct of research, research leadership and data governance

## CONCLUSION

The COVID-19 pandemic has come with its share challenges but has also opened opportunities and great lessons for the future. There is a clear need for junior faculty, emerging global leaders, and their mentors to prepare for unforeseen challenges. Team resilience, support, focus on goals, and capacity to adapt to changing learning culture are factors that could differentiate between success and failures in sustaining research and career development activities in unprecedented times.
